# Autoimmune encephalitis associated with anti-SOX1 autoantibodies in COVID-19: A case report

**DOI:** 10.1016/j.idcr.2025.e02220

**Published:** 2025-04-05

**Authors:** Peter Sabaka, Gabriela Timárová, Mohammad Dababseh, Eliška Marešová, Igor Straka

**Affiliations:** a Department of Infectology and Geographical Medicine, Faculty of Medicine, Comenius University in Bratislava, Bratislava Slovakia; b 2nd Department of Neurology, Faculty of Medicine, Comenius University in Bratislava, Bratislava, Slovakia

**Keywords:** COVID-19, Parainfectious encephalitis, Autoimmune encephalitis

## Abstract

Coronavirus disease 2019 (COVID-19) might be complicated by various non-respiratory conditions, including encephalitis. Encephalitis in COVID-19 represents a heterogenous group of diseases with variable aetiology. Autoimmune encephalitis (AIE) is the least common but one of the most severe causes of encephalopathy in COVID-19. AIE is a rare disease that is associated with different types of autoantibodies mostly directed against various neuronal antigens. Anti-Sry-like high mobility group box (SOX1) autoantibodies have been described in various clinical conditions, including Lambert-Eaton myasthenic syndrome, paraneoplastic cerebellar degeneration and rare cases of paraneoplastic AIE. We present the case of 28-year-old female patient with COVID-19 confirmed by the polymerase chain reaction (PCR) test. She was admitted with fever, headache, disorientation and new-onset refractory status epilepticus. Computed tomography and magnetic resonance imaging of the brain were unremarkable. Cerebrospinal fluid analysis showed pleocytosis, an increased total protein concentration and increased albumin and immunoglobulin G. Electroencephalography revealed findings suggestive of AIE. Serologic examination of antineuronal antibodies showed anti-SOX1 autoantibodies. A course of parenteral methylprednisolone and intravenous immunoglobulin led to rapid clinical improvement. The patient was discharged free of seizures as well as neurologic and psychiatric symptoms. After discharge, an oncologic screening was performed and ruled out a paraneoplastic aetiology.

## Introduction

Autoimmune encephalitis (AIE) is the most common form of non-infectious encephalitis in adults. It is characterised by the presence of encephalitis and meningoencephalitis in the absence of neuroinfection and typically by the presence of various autoantibodies [1]. The incidence is relatively low, but the disease is probably underdiagnosed and underreported [2]. While the clinical presentation is variable, the typical clinical sign is the presence of seizures. Epileptic seizures occur in 33 %-100 % of patients with AIE and often exhibit high resistance to antiseizure medication. Accumulation of epileptic seizures may lead to refractory status epilepticus, which represents a serious and life-threatening complication [3]. AIE has been recognised as one of the most common causes of new-onset refractory status epilepticus (NORSE) or febrile infection-related epilepsy syndrome (FIRES). Psychiatric symptoms including psychosis and hallucinations and other severe neurologic symptoms such as amnesia, movement disorders and loss of consciousness are also common [4]. The most common type of AIE is anti-*N*-methyl-D-aspartate (NMDA) receptor encephalitis, characterised by the presence of anti-NMDA receptor autoantibodies; however, there are numerous autoantibodies associated with AIE [1]. Anti-Sry-like high mobility group box 1 (SOX1) antibodies are associated with various clinical manifestations, including Lambert-Eaton myasthenic syndrome and paraneoplastic cerebellar degeneration. Anti-SOX1 antibodies are associated with rare cases of paraneoplastic encephalitis with NORSE [5], [6]. Coronavirus disease 2019 (COVID-19) is associated with the risk of developing various autoimmune diseases, notably rheumatoid arthritis, systemic lupus erythematosus, vasculitis and type 1 diabetes mellitus. The proposed mechanisms leading to autoimmunity in COVID-19 include antigen mimicry, bystander activation of immune cells, release of autoantigens from damaged tissue, viral superantigens and epitope spreading [7]. Encephalitis is a rare but potentially life-threatening complication of severe acute respiratory syndrome coronavirus 2 (SARS-CoV-2) infection. The pathomechanism leading to encephalitis in COVID-19 is not uniform. It includes direct viral invasion of the central nervous system and parainfectious and postinfectious autoimmune disease [8]. The exact incidence of COVID-19 associated with AIE is unknown, but it is regarded to be very rare [9]. We present a case of anti-SOX1-mediated AIE that presented as NORSE with acute COVID-19.

## The case

**Fig. 1:**
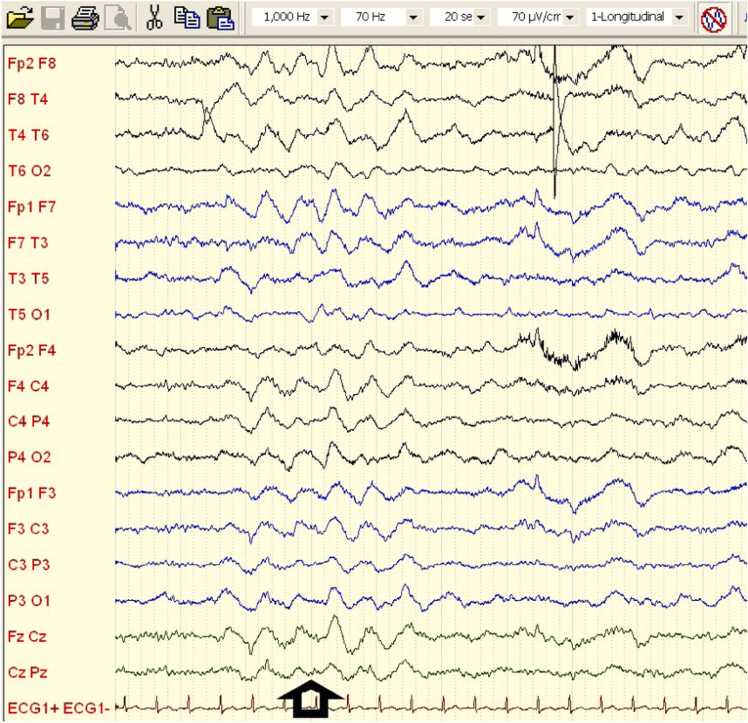
Encephalogram at the time of presentation. It shows generalised rhythmic delta with superimposed fast activity: extreme delta brush pattern (marked by black arrow).

**Fig. 2:**
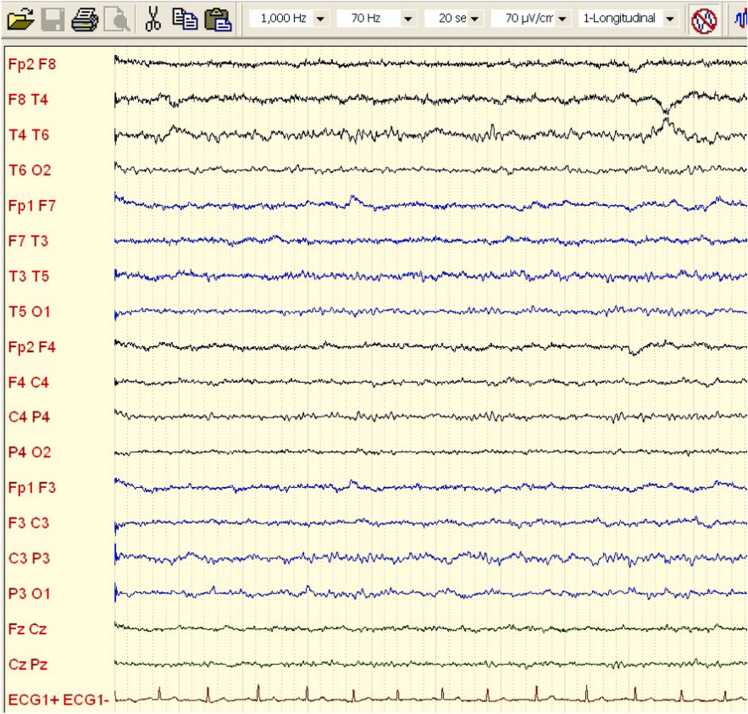
An electroencephalogram at the patient’s 4-month follow-up. It shows physiologic patterns.

A 28-year-old previously healthy Caucasian female (ethnic group: Slovak, West Slavic region) presented to the Emergency department of the Department of Infectology and Geographical Medicine, University Hospital Bratislava, with a history of headache, disorientation and tonic-clonic seizures. Two days before admission, she developed a low-grade fever, rhinitis and mild tiredness. A SARS-CoV-2 antigen self-test (nasal swab, lateral flow assay) was positive. One day before admission, she developed a mild headache. In the evening, according to her husband, she was mildly confused. During the night, she had at least five tonic-clonic seizures and was transferred to the emergency department via ambulance. At the emergency department, the seizures were terminated using 10 mg of intravenous diazepam. Subsequently, she was obnubilated and did not respond to commands. Neurologic examination revealed nuchal rigidity (opposition to flexion up to 5 cm) with no focal signs. Here body temperature was elevated to 39 °C, but otherwise, the physical examination was unremarkable. The biochemical examination of plasma including C-reactive protein and procalcitonin was unremarkable. A complete blood count revealed mild monocytosis (750 cells/mL; reference range: 100–700 cells/mL). Native computed tomography (CT) revealed no abnormalities. We performed a lumbar puncture, and an examination of the cerebrospinal fluid (CSF) revealed pleocytosis with 37 mononuclear cells/μL, 19 polymorphonuclear cells/μL and 56 erythrocytes/μL. The total protein concentration was elevated at 815 mg/L (reference range: 200–500 mg/l). The CSF albumin level (584 mg/L; reference range: 120/300 mg/:) and immunoglobulin G level (128 mg/L; reference range: 12–40 mg/L) were also elevated. The CSF glucose and lactate concentrations were unremarkable. Polymerase chain reaction (PCR) was negative for herpes simplex virus 1 and 2, varicella zoster virus, tick borne encephalitis, enterovirus, *Listeria monocytogenes*, *Neisseria meningitidis*, *Streptococcus pneumoniae*, *Haemophilus influenzae* and *Escherichia coli* nucleic acid in the CSF. The patient was admitted to the intensive care unit (ICU) and parenteral treatment with acyclovir 500 mg every 8 h and ceftriaxone 2 g every 12 h was started. In addition, antiseizure therapy with intravenous levetiracetam 2 g per day was started. During the first 2 days after admission, the patient remained soporous, she did not respond to commands and her Glasgow Coma Scale (GCS) score was 7 points (eye response: 2 points; motoric response: 4 points; verbal response: 1 point). The nonconvulsive epileptic state was suggested to be the cause of the quantitative impairment of consciousness. Therefore, the dose of levetiracetam was increased to 4 g per day. On day 3 after admission, the patient’s state of consciousness improved: she was awake, responded to commands and fully cooperated during diagnostic and therapeutic procedures. However, she was apathetic and did not provide verbal responses. Magnetic resonance imaging (MRI) with gadolinium contrast was conducted and revealed no abnormalities. SARS-CoV-2 PCR from a nasopharyngeal swab was positive (cycle threshold: 16 cycles), which confirmed the COVID-19 diagnosis. On day 4 after admission, the patient developed frequent focal tonic-clonic seizures limited to facial muscles and upper extremities (FAS) and focal seizures with impaired awareness (FIAS). The seizures were provoked by drinking and eating or touching the lips or perioral area. She also had up to five focal to bilateral tonic-clonic seizures (FBTCS) per day. She developed a severe headache; nausea and retching; and psychiatric symptoms including agitation, insomnia and repetitive and stereotypical behaviour. CT ruled out new focal neurologic involvement or significant brain oedema. By day 8 after admission, the frequency of FAS and FIAS had gradually increased up to 50 per day. At this time, MRI with gadolinium contrast was repeated; it again revealed no abnormalities. Lacosamide 400 mg per day was added and the levetiracetam dose was increased to 6 g per day with little to no effects on the seizure frequency or duration. Because the seizures were resistant to treatment, AIE was proposed and intravenous immunoglobulin (IVIG) at a dose of 20 g per day was introduced. Serum was drawn to examine antineuronal antibodies prior to commencing IVIG treatment. Electroencephalography (EEG) was conducted on day 10 after admission. It revealed generalised rhythmic delta frequency activity with superimposed fast activity known as extreme delta brush (Fig. 1). Examination of antineuronal antibodies associated with AIE revealed anti-SOX1 autoantibodies in the serum and cerebrospinal fluid. A course of parenteral methylprednisolone at a dose of 1 g per day was started. On the day 11 after admission – corresponding to the second day of the methylprednisolone course and the fourth day of the IVIG course – the frequency of seizures decreased to five per day. The patient’s nausea and retching resolved and her headache became milder. On day 13 after admission, the patient completed the 3-day course of methylprednisolone and the 5-day course of IVIG. Her clinical condition further improved in the following 5 days of hospitalisation. Her seizures and aphasia and psychiatric symptoms resolved completely. The patient was discharged on day 20 after admission. At the time of hospital discharge, she experienced only mild fatigue and a self-reported mild impairment of concentration. After discharge, the patient underwent oncologic screening. The gynaecologic examination, including gynaecologic and breast ultrasound and mammography, was negative. As the diagnosis of AIE was conclusive, the antineuronal antibody test was not repeated. The abdominal ultrasound and occult faecal bleeding test were unremarkable. The patient underwent whole-body [¹ ⁸F]fluorodeoxyglucose positron emission tomography; the findings were unremarkable and study found no sign of a tumour. Her out-patient follow-up at 4 months indicated complete remission of clinical symptoms, including a normal EEG (Fig. 2).

## Discussion

We have presented the first case of COVID-19-associated AIE with anti-SOX1 autoantibodies. COVID-19 is associated with the risk of developing a wide variety of autoimmune conditions, including AIE. In general, encephalitis is a rare complication of COVID-19, and AIE is regarded as a possible but infrequent cause of encephalitis in COVID-19. Retrospective studies focusing on the description of epidemiology of encephalitis in COVID-19 found an incidence of 0.1 %-0,2 %. However, the true incidence is difficult to estimate because of underreporting of both SARS-CoV-2 infections and cases of COVID-19-associated encephalitis [10], [11]. There is significant evidence that SARS-CoV-2 infection is a potent driver of autoimmunity. The risk of developing various autoimmune conditions, such as systemic lupus erythematosus, rheumatoid arthritis, vasculitis, inflammatory bowel disease and type 1 diabetes mellitus, has been reported to be associated with COVID-19 [12]. COVID-19 is associated with the presence of various autoantibodies [13]. The pathogenesis of autoimmune disorder development in COVID-19 has been the subject of intensive study. Antigen mimicry, bystander activation of lymphocytes, release of autoantigens from infected cells, viral superantigens and epitope spreading have been proposed as the possible mechanisms that drive autoimmune processes and the production of autoantibodies in patients with SARS-CoV-2 infection [7]. Antibodies against the N-protein of SARS-CoV-2 bind to various human proteins. Thus, SARS-CoV-2 antigenic mimicry might trigger the development of autoreactive lymphocytes [14]. Bystander activation of these autoreactive lymphocytes via extensive production of proinflammatory cytokines in the context of COVID-19 then drives their proliferation and the production of autoantibodies. It may also participate in the breakdown of immune tolerance of endogenous antigens [15]. Epitope spreading diversifies the epitopes recognised by the immune system from primary epitopes to secondary epitopes that are structurally similar and may decrease the threshold for immune tolerance of autoantigens. That may lead to formation of autoaggressive antibodies [16]. The hypothesis that antigen mimicry and epitope spreading are the important drivers of autoimmune reactions in patients with COVID-19 is supported by the finding that many human proteins contain epitopes with significant structural similarities to SARS-CoV-2 antigens [17]. A recent study summarised 42 cases of COVID-19-associated AIE.^18^ The most prevalent form of AIE in that case series was AIE with unknown antibodies (50 % of the cases), followed by anti-NMDA receptor AIE, limbic encephalitis and anti-myelin oligodendrocyte glycoprotein AIE. There were no cases of anti-SOX1 AIE. To our knowledge, COVID-19-associated AIE with the presence of anti-SOX1 autoantibodies has never been described in the available literature. AIE with anti-SOX autoantibodies has been reported in patients with oncologic diseases [5], [6]. In our patient, paraneoplastic aetiology was excluded based on thorough oncologic screening. Moreover, the disease manifested with acute COVID-19. Therefore, we conclude that the AIE in our patient developed in association with acute SARS-CoV-2 infection. Most cases of COVID-19-associated encephalitis are not autoimmune in nature and may be caused by other mechanisms [18]. We concluded that the encephalitis in our patient had an autoimmune aetiology because she presented the typical AIE clinical signs – new-onset seizures, fever, profound psychiatric abnormalities, dysarthria, CSF pleocytosis and typical EEG alterations – and positivity of AIE-associated autoantibodies [19]. Moreover, we excluded common infectious causes of encephalitis.

The clinical and laboratory presentation of COVID-19-associated AIE is similar to paraneoplastic or other cases of AIE not associated with COVID-19. AIE in COVID-19 typically manifests with the new onset of tonic-clonic seizures and NORSE. Patients often exhibit various mental and behavioural disturbances such as cognitive decline, abnormal behaviour, delusions of persecution and hallucinations. Laboratory and radiologic findings are usually unspecific. The most prevalent CSF abnormalities are moderately elevated proteins and lymphocytic and monocytic pleocytosis [18]. However, about 30 % of cases of AIE lack CSF pleocytosis [20]. MRI of the brain might be unremarkable or might show signal hyperintensity in various regions of white or grey matter [18], [21]. In a large retrospective multicentre study, about 40 % of patients with AIE had no signal abnormalities on MRI [20]. EEG abnormalities are common in AIE. Generalised delta frequency activity is present in about one third of patients with AIE. Generalised rhythmic delta activity with superimposed fast activity, known as extreme delta brush, is associated with but not limited to anti-NMDA encephalitis [22]. Our patient presented with extreme delta brush on EEG, which supported the diagnosis of AIE. Once the diagnosis of COVID-19-associated AIE is established, the prognosis is favourable. Indeed, almost 70 % of patients experience complete recovery [18]. The first-line treatment is immunosuppressive therapy consisting of high-dose IVIG and systemic corticosteroids. Plasmapheresis might be used instead of IVIG. Second-line immunosuppressive therapy is recommended if the first-line therapy fails; it may involve rituximab and cyclosporine [23]. Our patient responded to the first-line therapy of IVIG and methylprednisolone.

## Conclusion

AIE is rare complication of COVID-19. Approximately half of the cases have an unknown aetiology and most of the remaining show the presence of anti-NMDA receptor antibodies. To the best of our knowledge, there has not been a previous report of AIE associated with anti-SOX1 autoantibodies in the context of acute SARS-CoV-2 infection. AIE with NORSE is potentially life threatening condition and rapid establishing of diagnosis is crucial for appropriate disease management. The recognition of typical clinical course and subsequent EEG and serum autoantibody analysis are critical for the correct diagnosis and early introduction of life saving immunomodulation therapy. First-line immunotherapy consists of corticosteroids and IVIG. It is important to consider the possible AIE within in patients with COVID-19, meningoencephalitis and epileptic seizures because of its life threatening nature and treatability.

## Final statement

All authors read the revised manuscript and accepted all the changes to the original manuscript.

## Author agreement

This statement is to certify that all authors have seen and approved the manuscript being submitted, have contributed significantly to the work, attest to the validity and legitimacy of the data and its interpretation, and agree to its submission to the *IDCases.* We attest that the article is the Authors' original work, has not received prior publication and is not under consideration for publication elsewhere. On behalf of all Co-Authors, the corresponding Author shall bear full responsibility for the submission. Any changes to the list of authors, including changes in order, additions or removals will require the submission of a new author agreement form approved and signed by all the original and added submitting authors.

## CRediT authorship contribution statement

**Straka Igor:** Conceptualization. **Sabaka Peter:** Writing – original draft, Conceptualization. **Timárová Gabriela:** Writing – original draft, Visualization. **Dababseh Mohammad:** Writing – original draft. **Marešová Eliška:** Writing – original draft.

## Consent

This case study has been conducted in accordance with the Declaration of Helsinki and approved by the local ethics committee of the University Hospital Bratislava. Written informed consent was obtained from the patient for publication of this case report and the accompanying images. A copy of the written consent is available for review by the Editor-in Chief of this journal on request.

## Funding

This case study did not receive any specific grant from funding agencies in the public, commercial or not-for-profit sectors.

## Declaration of Generative AI and AI-assisted technologies in the writing process

No generative AI and AI-assisted technologies were used in the writing process of this manuscript.

## Declaration of Competing Interest

The authors declare that they have no known competing financial interests or personal relationships that could have appeared to influence the work reported in this paper.
